# On the Two Movements of the Brain,—the Respiratory and Arterial

**Published:** 1849-02

**Authors:** 


*On the two movements of the Brain,—the Respiratory and Arterial.*—There are two movements of the cerebral substance, the one which corresponds with the dilatation of the arteries, the other to the movements of respiration.

1. *Respiratory movement of the brain.*—The respiratory movement of the brain has been the subject of memoirs by Haller, and Lamare, &c.; the latter also observed a correspondence between these movements and the flux and reflux of the venous blood. During expiration, the blood flows back from the superior vena cava into the cerebral sinuses, and the brain becomes distended. In inspiration, on the contrary, the blood retires from the brain into the jugulars, and the organ diminishes in bulk. M. Flourens has also investigated the subject, and finds that the blood, which, by its reflux distends the brain, is derived, not as the others suppose, from the jugulars, but from the two great vertebral sinuses.

2. *Arterial movements of the brain.*—In his work on the cerebro-spinal movements, M. Flourens performed his experiments on rabbits, the blood-vessels of which animal were too small to communicate a movement to the cerebral mass, and he accordingly denied its existence. When, however, he repeated his experiments on larger animals, as dogs, he perceived this class of movements to be indubitable. Having trephined the frontal bone, without injuring the dura mater, he distinctly saw an alternate pulsation of the brain coincident with the systole and diastole of the blood-vessels. In the first dog he counted 68 movements of the brain in a minute, and 68 pulsations of the crural artery; the respiratory thoracic movements being only 24. In a second dog he saw 80 pulsations of the brain and crural artery, to 20 respiratory movements. In both there was a cerebral movement also in correspondence with the respiratory, being in the first dog 24 in number, in the second 20. Those movements were quite distinct from the arterial, and strictly proportionate to the intensity of the thoracic respirations.—*Prov. Med. and Surg. Jour. from Gaz. Med.*

